# Traditional knowledge of wild edible plants with special emphasis on medicinal uses in Southern Shan State, Myanmar

**DOI:** 10.1186/s13002-018-0248-1

**Published:** 2018-07-17

**Authors:** Thant Shin, Kazumi Fujikawa, Aung Zaw Moe, Hiroshi Uchiyama

**Affiliations:** 1 College of Bioresource Sciences, Nihon University, 1866 Kameino, Fujisawa, Kanagawa 252-0880 Japan; 2 Forest Department, Ministry of Natural Resource and Environmental Conservation, Forest Research Institute, Yezin, Naypyitaw Myanmar; 3 Kochi Prefectural Makino Botanical Garden, 4200-6, Godaisan, Kochi, 781-8125 Japan; 4 Ministry of Natural Resource and Environmental Conservation, Forest Research Institute, Yezin, Naypyitaw Myanmar

**Keywords:** DNA barcode, Ethnobotany, Myanmar, Medicinal plants, Wild edible plants

## Abstract

**Background:**

Myanmar is one of the hotspots of biodiversity and is a rapidly developing country. Performing floristic research in Myanmar is an urgent issue, and ethnobotanical studies of wild edible plants (WEPs) will provide new information on natural plant resources.

**Method:**

Ethnobotanical data were collected in three villages with different historical backgrounds in Southern Shan State, Myanmar. A total of 19 key informants were interviewed, and specimens were collected in the fields with the participation of key informants in June–July 2015. Group discussions were organized during 2016 and 2017 to reinforce the information on use of WEPs. DNA barcoding was used to facilitate species identification.

**Results:**

A total of 83 species from 44 families of angiosperms were recorded as WEPs. Most of the species were used as wild vegetables (47 species), followed by fruits and nuts (31 species). Eighteen WEPs were consumed as medicinal foods. Differences in use of WEPs between the communities of the villages were observed. The age class of 30–39 years was more familiar with the environments where they could collect WEPs and had more knowledge of WEPs than did the older groups. The use of *Elaeocarpus floribundus* as an edible oil is a very interesting tradition.

**Conclusion:**

WEPs play an important role in the livelihood of local communities. The indigenous society has maintained traditional knowledge of the WEPs. Historical background, land use system and surrounding vegetation could have effects on the variation in the traditional uses of WEPs. Increasing awareness of the importance of WEPs will encourage the conservation of traditional knowledge of indigenous populations.

## Background

Wild edible plants (WEPs) are defined as plant species collected in the wild to be consumed as food or drink. Although important nutrients for humans are available from WEPs, it was argued that the use of WEPs is decreasing in urban-style cooking [1, 2]. Being an important source of energy and micronutrients, WEPs can increase the diversification of human diets [3, 4]. WEPs were also important food sources during famine, when the normal food supply was disrupted [2]. Currently, humans focus on a limited number of plant species for staple food, neglecting the importance and usefulness of WEPs. This restriction can lead to global food shortage and loss of the knowledge about WEPs [5, 6]. Documentation of WEPs is important for the identification of food sources from the surrounding environment, and WEPs are serving as gene pools for genetic improvement of crops to achieve higher productivity, disease resistance and compatibility with global climate change [7]. WEPs also have the potential to be developed into new crops through domestication [8]. Moreover, the nutritional and medicinal properties of WEPs are increasingly recognized [9–12]. The traditional uses of plant resources and the lifestyles of rural communities have changed in accordance with the switch from subsistence farming/hunting and gathering to profit-oriented agricultural systems [9]. The local food tradition is a kind of cultural expression, and a loss of traditional knowledge of WEPs implies a loss of cultural identity [13].

Myanmar is one of the hotspots of biodiversity [14, 15] and is a rapidly developing country. Describing the flora of Myanmar is an urgent issue, and ethnobotanical studies will provide new information on natural plant resources [16, 17]. A total of 135 ethnic groups are officially recognized in Myanmar [18]. These indigenous people regard natural resources as essential to their culture and well-being [19], but they are not truly interested in the conservation of natural resources. Furthermore, local communities are rarely allowed to participate in decision-making processes concerning the impact of ecosystem changes, and their dependence on plant resources is not adequately considered in formulating the strategies of rural development [20]. In Myanmar, quite a few ethnobotanical studies have been carried out mostly emphasizing medicinal and financial value [21–24].

**Fig. 1:**
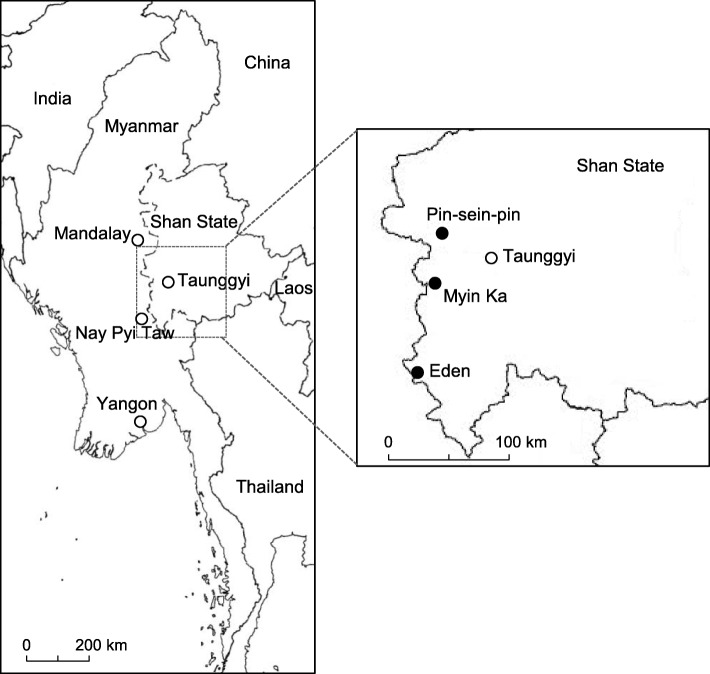
Map of study area location

Field research was carried out in three villages, each with different historical backgrounds, namely Eden, Myin Ka, and Pin-sein-pin, located in the western part of Southern Shan State (Table 1 and Fig. 1). Southern Shan State is situated on the Shan Plateau, which rises to the east from the central basin of Myanmar and occupies the eastern half of the country. The average elevation of the plateau is approximately 900 m. The average annual precipitation is between 1900 mm and 2000 mm, and the average daily temperature is 22 °C [25]. The ethnic groups residing in the study area are *Danu, Taung-yoe, Shan, Bamar, Pao, Kayan* and *Kayin.* The majority of people in the villages of Myin Ka and Pin-sein-pin are *Danu* and *Taung-yoe*. The major ethnic group in Eden village is *Kayan*. According to the folklore of the villagers, the old village of Myin Ka was established during the *Bagan* Era (9th–13th centuries) one mile away from the current village location. In the late nineteenth century, the old village was destroyed because of ethnic conflicts in the area and was moved to the current location. The villagers of Eden migrated from Kaya State because of civil war in their former area and established the village in 1990.

**Table 1:** Basic information of the three villages studied

	Myin Ka	Pin-sein-pin	Eden
Establishment	Before late nineteenth century (Moved from nearby old village, one mile away).	Early nineteenth century	1990
Location	N: 20°34′54.3″, E: 96°34′52.7″	N: 20°58′54.3″, E: 96°37′54.9″	N: 19°55′31.9″, E: 96°25′58.1″
Above sea level	1422 m	1736 m	353 m
Habitat	Evergreen forest	Evergreen forest	Deciduous forest
Linear distance to the nearest town	10 km to Kalaw, Shan State	8 km to Pindaya, Shan State	40 km to Tatkone, Mandalay Division
Population/Households	563/140	535/127	410/68
Ethnic Group	Majority: Taung-yoe, Danu Minority: Bamar, Shan	Majority: Danu Minority: Bamar, Pao	Majority: Kayan Minority: Kayin, Bamar
Religion	Buddhism	Buddhism	Christian and Buddhism
Public Facility	One library, one primary school, one buddhist monastery	One primary school, one library, one kindergarten	One middle school, one church, one monastery, one kindergarten
Subsistence	Rice cultivation (ca. 60 acres) Cultivation of vegetables and fruits: ginger, cabbage, cauliflower, egg plants, tomato, chili, chayote, carrot, orange, avocado, pear (ca 150 acres)	Plantation of tea leaves, Ju, pea, cabbage, potato	Cultivation of turmeric, chili, banana, hill rice Collection of bamboo shoots and other forest products
Home garden products	Ginger, orange, avocado, pear	Tea leaves	Mango, banana, jack fruit
Live stock	Buffalo, chicken, cattle, pig	Cattle, chicken	Pig, chicken
Drinking water	Natural springs (connected with pipe line)	Collection of rain water with collection tanks	Natural springs (connected with pipe line)
Electricity	From national grid	No electric supply	Small hydropower generators constructed on the stream

This study focused on the local communities in Southern Shan State to document their traditional knowledge of and practices involving WEPs. In the present study, a DNA barcoding technique was used to facilitate species identification. DNA barcoding is a microgenomic identification system in which a short, standard DNA region that is universally present in the target lineages is analyzed [26, 27]. With the help of DNA barcoding techniques, species identification can be facilitated in the ethnobotanical studies [28, 29] and floristic studies of unexploited tropical regions [30]. A total of 83 species from 44 families of angiosperms have been documented as WEPs in the study area. The indigenous societies, who have been living in the area for a long time, could have a considerable amount of traditional knowledge of WEPs.

## Methods

### Interviews and collection of plant specimens

First, meetings were organized with villagers, and the objectives of the research were explained. Consent was obtained before carrying out the ethnobotanical survey. The ethical guidelines of the International Society of Ethnobiology were strictly obeyed [31].

The ethnobotanical data were first collected from 19 key informants who were selected for their reputation of being specialists in the use of WEPs. The information on WEPs was gathered from interviews and fieldwork. The ages of the key informants ranged from 30 to 57 years, and one informant was female. All key informants were farmers. The field surveys were carried out in June–July 2015, and voucher specimens and the leaves for DNA analysis were collected. During the field surveys, the vernacular name, growth habitat and folk use of each species were recorded. Interviews and field sampling were carried out with one individual informant at a time to avoid the incorporation of the knowledge from other villagers. Voucher specimens were deposited in the herbaria of the Makino Botanical Garden (MBK), Japan, and the Forest Research Institute (RAF), Myanmar.

After gathering ethnobotanical data from the key informants, group discussions were organized again in 2106 and 2017. In addition to the 19 key informants, 23 other informants were invited for the group discussion. These 23 informants were selected for their willingness to participate in this study, ability to work together in the field, and reputation for being knowledgeable on the use of WEPs. Therefore, a total of 42 villagers participated in this project, including 5 female informants. A total of 13, 12 and 17 informants from the villages of Eden, Myin Ka and Pin-sein-pin, respectively, participated in the group discussion. The age of informants ranged from 23 to 82 years, and the group included 37 farmers, one small shop owner, one forester and three herbalists. We also gathered information on WEPs with a group of informants by using the “walk-in-the-wood” method [32].

### DNA barcoding and species identification

Total DNA was extracted from dried leaf tissue by the CTAB method. Approximately 20 mg of dried leaf tissue was ground in a 2 mL microcentrifuge tube using TissueLyser (Qiagen, Hilden, Germany). The sample was incubated with 2xCTAB solution at 65 °C for 30 min. After chloroform (chloroform:isoamylalcohol = 24:1) treatment for 30 min, the mixture was centrifuged for 10 min at 3000 rpm. The aqueous layer was transferred to a new 1.5 mL microcentrifuge tube, and then the DNA was precipitated with ethanol. The pellet was dissolved with TE buffer. PCR amplification of the *rbcL* region followed the procedure of the CBOL Plant Working Group [33]. rbcLa_F and rbcLa_R were used as primers. For the PCR, GoTaq Green Master Mix (Promega, Madison, WI, USA) was used. Amplification was performed in a TaKaRa PCR Thermal Cycler Dice Touch (Takara, Kusatsu, Japan) programmed for one cycle of 95 °C for 5 min; 30 cycles of 95 °C for 30 s, 55 °C for 30 s, and 72 °C for 1 min; and one cycle of 72 °C for 5 min. The PCR products were resolved by electrophoresis in 1.0% agarose gels stained with GelGreen (Wako, Osaka, Japan). DNA fragments were recovered using the Wizard SV Gel and PCR Clean-up System (Promega, Madison, WI, USA) according to the manufacturer’s instructions. Cycle sequencing was performed using a BigDye Terminator v3.1 Cycle Sequencing Kit and an ABI 3130 Genetic Analyzer (Thermo Fisher Scientific, Waltham, MA, USA).

The Basic Local Alignment Search Tool (BLAST) program was used as a tool to search for similar DNA sequences from the database of the National Center for Biotechnological Information (NCBI). Among the compared sequences, the species with the most similar sequences were used to guide species identification. The final identification of specimens was determined by cross referencing with specimens housed at MBK, the type specimen images available on the JSTORE Global Plants website (https://plants.jstor.org), and the online herbarium catalog of the Royal Botanic Gardens, Kew (http://apps.kew.org/herbcat/navigator.do). The plant descriptions in the Flora of China and Flora of Thailand were consulted. The scientific names of species were assigned in accordance with The Plant List (http://www.theplantlist.org). The uses of WEPs and medicinal plants were classified into categories according to the standards developed by the Royal Botanic Gardens, Kew [34].

## Results

### Diversity of WEPs in the study area

A total of 153 specimens were collected as WEPs from the three villages in this study. Of these, 138 specimens were identified to the species level and consisted of 83 species. Araceae, Fabaceae and Moraceae were the major families with the largest number of species (5 species each). Zingiberaceae, Asteraceae, Myrtaceae and Rutaceae included four species. Ethnobotanical data such as vernacular names, collection sites and folk uses are listed in Table 2. Among the 83 species, most are indigenous in Myanmar, but some of them originated outside of Asia: *Annona cherimola*, *Casimiroa* cf. *edulis*, *Coffea arabica* and *Psidium guajava* escaped from plantations; and *Alternanthera philoxeroides*, *Bassia scoparia*, *Bidens biternata*, *Crassocephalum rubens*, *Marah macrocarpa*, *Oxalis latifolia*, *Physalis angulata*, *Physalis pubescens* and *Solanum torvum* are naturalized.

**Table 2:** List of identified wild edible plants used by villagers in this study

Taxon	Village^a^ and vernacular name	Use	Voucher number (Collection site^a^)
Amaranthaceae			
*Alternanthera philoxeroides* (Mart.) Griseb.	M, P: Shwe-kana-phot, Ka-na-phot	Food and Medicine: shoot for salad, consumed for body swollen	TS0366 (P)
*Amaranthus viridis* L.	M: Hin-nu-nwe	Food: tender leaves as vegetable	TS0020 (M)
*Bassia scoparia* (L.) A.J.Scott	M: Ta-byat-se	Food: shoots and tender leaves as vegetable	TS0019 (M)
Anacardiaceae			
*Mangifera sylvatica* Roxb.	E: Taw-tha-yat	Food and Construction: fresh fruits pounded as salad with other ingredients, sliced fresh fruits for sour taste	TS0809 (E)
Annonaceae			
*Annona cherimola* Mill.	M, P: Aw-le	Food: fruits edible	TS0043 (M)
Apiaceae			
*Centella asiatica* (L.) Urb.	E, M, P: Myin-kwar	Food and Medicine: tender leaves as salad, paste of leaves prepared lotion for sore throat, cold infusion of leaves as eye drop	TS0165, TS0293 (M)
*Oenanthe* cf. *javanica* (Blume) DC.	M: Za-lae	Food: tender leaves as salad, cooked as traditional curry	TS0138, TS0166 (M)
Apocynaceae			
*Telosma cordata* (Burm. f.) Merr.	E, M, P: Gwe-tauk	Food and Medicine: tender leaves for soup with chicken, consumed food as medicine for alcohol dependence	TS0190 (M)
Araceae			
*Amorphophallus* cf. *muelleri* Blume	E, M, P: Wa-u	Food: stem and bulb as vegetable: young stem cooked as vegetable, bulb boiled and grounded to make konjac (Wa-u)	TS0728/1 (E)
*Amorphophallus purpurascens* Kurz ex Hook.f.	E, M, P: Wa-u	Food: stem and bulb as vegetable: young stem cooked as vegetable, bulb boiled and grounded to make konjac (Wa-u)	Thant Shin (abbreviate as TS hereafter) 0618 (P)
*Arisaema erubescens* (Wall.) Schott	M, P: Wa-u-pho	Food and Medicine: stem and bulb as vegetables, bulb boiled and eaten for constipation	TS0284 (M); TS0599 (P)
*Colocasia esculenta* (L.) Schott	E: Pain-ga-nan; M, P: Pai	Food and Medicine: petiole fermented, petiole as vegetable, petiole prepared soup with *Kin-pun-chin, Sue-pote and Zayit*; sap used externally for allergy caused by insects	TS 0040, S0137, TS0277 (M)
*Lasia spinosa* (L.) Thwaites	E, M: Za-yit	Food: tender shoot cooked as soup, cooked with fish, boiled shoot as salad with fish paste	TS0757 (E)
Araliaceae			
*Macropanax dispermus* (Blume) Kuntze	P: Tha-yat-kin, Ka-la-kin	Food, Construction, Fuelwood and Medicine: shoot as vegetable dish and salad, food consumed for retention of gasses in bowel	TS0590, TS0619, TS0645 (P)
Asteraceae			
*Bidens biternata* (Lour.) Merr. & Sherff	P: Hlan-kwa	Food: shoot as vegetable	TS0563 (P)
*Crassocephalum rubens* (Juss. ex Jacq.) S.Moore	E: Nu-su; P: Taw-bi-zat	Food: shoot as vegetable, tender leaves for salad	TS00684 (P); TS0776 (E)
*Dichrocephala integrifolia* (L.f.) Kuntze	M: Sein-zar-myat-lone	Food and Medicine: shoots and tender leaves for soup, food consumed as medicine at postpartum period for mother	TS0038 (M)
*Laggera alata* Nanth.	M, P: La-thar-ba-pyin	Food and Medicine: shoots fried with eggs, good for swollen body, the whole plant as wristband for back pain, leaves extract used over knife injuries	TS0078 (M)
Bignoniaceae			
*Markhamia stipulata* (Wall.) Seem.	E: Ma-lwa	Food and Construction: boiled flower as salad with fish paste, fried as vegetable	TS0735 (E)
*Oroxylum indicum* (L.) Kurz	E, M, P: Kyaung-shar	Food and Medicine: flowers and fruits for vegetable dish, fruits as salad, farmented fruit, boiled and pounded fruits as salad with other ingredients, young leaves prepared salad, and consumed orally for tinnitus	TS0105 (M); TS0743 (E)
Burseraceae			
*Protium serratum* (Wall. ex Colebr.) Engl.	E: Kadi	Food and Construction: fruits eaten fresh	TS0704 (E)
Celastraceae			
*Celastrus paniculatus* Willd.	M, P: Taung-bort-lu-lin	Food and Medicine: tender leaves prepared for soup, diet food for healthy life	TS0230 (M)
Combretaceae			
*Terminalia bellirica* (Gaertn.) Roxb.	E, M, P: Thit-seint	Food: seeds eaten fresh	TS0702 (E)
Costaceae			
*Cheilocostus speciosus* (J.Koenig) C.D.Specht	E, M: Pha-lan-taung-hwa	Food and Medicine: shoot fried with vegetable oil and other ingredients, cooked for soup, cooked with bamboo shoot and meat, decotion of whole plant taken orally for dysentery	TS0037 (M); TS0732 (E)
Cucurbitaceae			
*Marah macrocarpa* (Greene) Greene	M: Kin-mon-tee	Food: fruits as vegetable	TS0094 (M)
*Momordica subangulata* Blume	M: Taw-hin-khar	Food and Medicine: fruits and leaves as vegetable, consumed as appetizer	TS0114, TS0276 (M)
Ebenaceae			
*Diospyros kaki* L.f.	M: Tae, Tel; P: Tel	Food: ripe fruits edible	TS0142 (M)
Elaeagnaceae			
*Elaeagnus griffithii* Servettaz	P: Mat-lwat, Myat-lu	Food: ripe fruits edible	TS0359, TS0480 (P)
Elaeocarpaceae			
*Elaeocarpus floribundus* Blume	M: Sein-sar-blue-pan	Food and Fuellwood: seeds used to extract edible oil, seed edible	TS0183 (M)
*Elaeocarpus stipularis var. siamensis* (Craib) Coode.	M: Sein-se-ba-lu	Food: ripe fruits edible	TS034 (M)
Fabaceae			
*Acacia concinna* (Willd.) DC.	E, M; Kin-mon-chin	Food and Shampoo: decoction of fruits for shampoo, tender leaves prepared soup with bean, prepared salad, fried with fish paste	TS0139 (M)
*Acacia pennata* subsp*. kerrii* I.C.Nielsen	E: Sue-pote, Sue-pote-kyi; M, P: Sue-pote	Food: tender leaves cooked as soup, cooked with fish, cook with meat, fried with egg, vegetable dish	TS0736 (E)
*Archidendron jiringa* (Jack) I.C.Nielsen	E, M, P: Da-nyin	Food: boiled seeds	TS0710, TS0811 (E)
*Bauhinia purpurea* L.	E: Swe-daw; P: Kha-lat	Food: tender leaves as soup with potato	TS0419 (P); TS0856 (E)
*Bauhinia variegata* L.	P: Kha-la	Food: leaves as salad. Seed edible as pulses	TS0629 (P)
Fagaceae			
*Lithocarpus lindleyanus* (Wall. ex A.DC.) A.Camus	M: Thit-al-sein	Food and Constructiion: roasted seed edible	TS0240 (M)
Lamiaceae			
*Rotheca serrata* (L.) Steane & Mabb.	M: Yin-byar; P: Hin-byar, Hin-khar	Food and Medicine: tender leaves and flowers as vegetable and salad, young leaves consumed as salad for retention of gasses in bowel, and diarrhea, cream of rhizome used as lotion on abdomen for retention of gasses in bowel, roots fermented together with jaggery and consumed orally for loss of sleep	TS0005, TS0082, TS0209, TS0279 (M); TS0387, TS00494, TS0591, TS0606 (P)
Lauraceae			
*Cinnamomum tamala* (Buch.-Ham.) T.Nees & Eberm.	P: Thit-kya-poe	Food and Medicine: leaf for spice in traditional curry, dry bark powder consumed orally as blood tonic, dry leaf powder used as inhalant at postpartum period for mother	TS0678 (P)
*Laurus* cf. *nobilis* L.	M, P: Lae-lu	Food: leaves for spices	TS0483 (P)
Lythraceae			
*Duabanga grandiflora* (DC.) Walp.	E: Ga-zaw	Food and Construction: fruits edible	TS00716 (E)
Melastomataceae			
*Osbeckia nepalensis* Hook. f.	P: Shar-pyar-tee	Food: fruits edible	TS00631(P)
Moraceae			
*Ficus auriculata* Lour.	M: Ka-ohn, Kaung-oat-tee, Tha-phan; P: Pha-owl	Food: ripe fruits edible	TS0042, TS0110, TS0182 (M); TS0461(P)
*Ficus racemosa* L.	E, M, P: Tha-phan	Food: ripe fruit edible, fresh fruits soaked in salty water, pounded leaves as paste, tender leaves as salad, leaves cooked with potato	TS00148 (M)
*Ficus semicordata* Buch.-Ham. ex Sm.	E, M, P: Ka-dut; P: Tha-phan	Food and Fuelwood: ripe fruit edible, fresh fruits fermented for food, tender leaves for soup	TS0071, TS00131, TS00172 (M); TS0474 (P); TS0788 (E)
*Ficus virens* Aiton	M, P: Nyaung-chin	Food: leaves and shoots for soup and salad	TS0140 (M)
*Maclura fruticosa* (Roxb.) Corner	P: Sue-sein	Food: shoot as vegetable for soup and salad	TS0478 (P)
Myricaceae			
*Myrica esculenta* Buch.-Ham. ex D. Don	M: Kata-pho	Food: ripe fruits edible	TS0058, TS0109, TS0180 (M)
Myrtaceae			
*Psidium guajava* L.	E: Mar-la-kar	Food: fruits eaten fresh	TS0760 (E)
*Syzygium cumini* (L.) Skeels	E, M, P: Tha-pyay	Food and Construction: ripe fruits edible	TS0215 (M)
*Syzygium oblatum* (Roxb.) Wall. ex A.M.Cowan & Cowan	E, M, P: Tha-pyay	Food and Construction: ripe fruits edible	TS0253 (M)
*Syzygium pycnanthum* Merr. & L.M.Perry	E, M, P: Tha-pyay	Food, Construction and Fuelwood: ripe fruits edible	TS0104 (M); TS0615 (P)
Oleaceae			
*Anacolosa clarkii* Pierre	M: Tay-pin	Food: fruits edible	TS0259 (M)
Oxalidaceae			
*Oxalis latifolia* Kunth	M: Mu-chin	Food: children eat all parts of plants	TS0024 (M)
Phyllanthaceae			
*Antidesma acidum* Retz.	E: Kim-ma-lin	Food: fruits and leaves as vegetable	TS0795 (E)
*Bischofia javanica* Blume	M: Yae-pa-done	Food: shoots for salad, young leaves as vegetable	TS0041, TS0074 (M)
*Phyllanthus emblica* L.	E: Zepyu; M: Se-sar; P: Se-shar	Food, Fuelwood and Medicine: fruits pounded as salad, boiled fruits pounded and prepared salad, consumed as food for hypertension, fruits eaten fresh for over bleeding, fruits roasted and consumed as food for cough	TS0033, TS0052, TS00213, TS0185, TS00280 (M); TS0351, TS0670 (P); TS0806 (E)
Plantaginaceae			
*Plantago major* L.	M: A kyaw-baung-tha-thaung	Food and Medicine: tender leaf for salad, leaf heated on fire to become wilted, and touch on abscess and boils, paste of leaves used as bandage for broken legs, young leaves prepared as salad, consumed as food for toxemia and hypertension; paste of entire plant used over abscess	TS0089 (M)
Primulaceae			
*Embelia ribes* Burm.f.	M, P: Kan-pa-lar	Food: leaves as salad and soup, tender leaves and buds boiled and eaten with fish sauce	TS0175 (M)
Rhamnaceae			
*Ziziphus incurva* Roxb.	P: Sue-kauk	Food: fruits edible	TS0688 (P)
Rosaceae			
*Docynia indica* (Wall.) Decne.	M, P: Pin-sein	Food and Fuelwood: fruits eaten fresh, cooked as soup, pounded with chili as side dish	TS0032, TS0133, TS0168, TS0204 (M); TS0399, TS0429, TS0513, TS 0588, TS0638 (P)
Rubiaceae			
*Coffea arabica* L*.*	P: Coffee	Food: roasted seed for coffee	TS0519 (P)
*Wendlandia budleioides* Wall. ex Wight & Arn.	M, P: Thit-ne	Food and Construction: tender leaves for salad	TS0085 (M)
*Wendlandia tinctoria* (Roxb.) DC.	M, P: Thit-ni	Food and Fuelwood: tender leaves as vegetable	TS0176 (M)
Rutaceae			
*Casimiroa* cf. *edulis* La Llave	M, P: Tha-gyar-tee	Food: fruits edible	TS0044 (M)
*Clausena excavata* Burm.f.	M: Pyin-thaw-sein	Food: tender leaves as raw salad	TS0069 (M)
*Murraya koenigii* (L.) Spreng.	P: Pyin-taw-thein	Food: tender leaves as raw salad	TS0543 (P)
*Zanthoxylum armatum* DC.	M: Mike-cup	Food: tender leaves ingredient to beef curry	TS0030 (M)
Salicaceae			
*Casearia graveolens* Dalzell	E: Phan-khar	Food and Construction: fruits eaten fresh	TS0801(E)
Sapindaceae			
*Choerospondias axillaris* (Roxb.) B.L.Burtt & A.W.Hill	E: Del-cline	Food: fruits edible	TS0765 (E)
*Dimocarpus fumatus* (Blume) Leenh.	E: Taw-kyat-mauk	Food: fipe fruits edible	TS0803 (E)
*Spondias pinnata* (L. f.) Kurz	E: Gwe	Food: fruits eaten fresh	TS0812 (E)
Schoepfiaceae			
*Schoepfia fragrans* Wall.	P: Byauk-ole-kyi	Food: fruits edible	TS0610 (P)
Smilacaceae			
*Smilax gagnepainii* T.Koyama	M: Sue-yit-sein; P: Sue-yit	Food: shoots fried as vegetable, cooked soup, green salad	TS0617 (P)
Solanaceae			
*Physalis angulata* L.	E: no name	Food: fruits eaten fresh	TS0756 (E)
*Physalis pubescens* L*.*	P: Taw-kha-yan-chin	Food: fruits eaten fresh	TS0477 (P)
*Solanum torvum* Sw*.*	E, M: Kha-yan-ka-zot; P: Ka-zot	Food: fruits as vegetable	TS0186 (M); TS0737 (E)
Theaceae			
*Camellia taliensis* (W.W.Sm.) Melch.	M, P: Taw-la-phat	Food: tender leaves for salad	TS0112 (M); TS0339 (P)
*Schima wallichii* Choisy	M: Thit-yar	Food and Constructiion: shoots as vegetable	TS0086 (M)
Urticaceae			
*Dendrocnide basirotunda* (C.Y.Wu) Chew	E: Tha-phan	Food: ripe fruits edible	TS0780 (E)
Vitaceae			
*Leea indica* (Burm. f.) Merr.	E: no name; M: Pait-chin	Food: shoot as vegetable	TS0226 (M); TS0805 (E)
Zingiberaceae			
*Alpinia nigra* (Gaertn.) Burtt	M: Gon-min	Food: pith as vegetable	TS0278 (M)
*Curcuma* cf. *amada* Roxb	M: Ba-thae-kaw	Food and Medicine: dry powder of rhizome consumed orally for flatulence, pounded fresh rhizome as spice in traditional curry, rhizome sliced and dry and made powder, and used as spice in curry	TS0068 (M); TS0727 (E)
*Curcuma aromatica* Salisb.	M: Mar-lar-pu	Food and Medicine: buds as vegetable, paste of rhizome used externally over injury	TS0210/1 (M)

^a^: E, Eden; M, Myin Ka; P, Pin-sein-pin

**Fig. 2:**
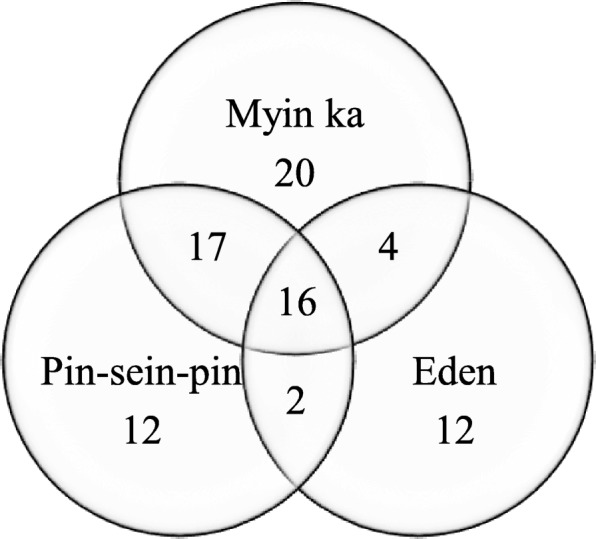
Number of WEP species used in each village

In total, 34, 57 and 47 species were used as WEPs in the villages of Eden, Myin Ka and Pin-sein-pin, respectively (Fig. 2). Although most of the WEPs were used with a single vernacular name within a village, eight species had two or three local names. *Physalis angulata* and *Leea indica* were used without local names in Eden village. Two species of *Amorphophallus* and three species of *Syzygium* were used with the same local name in all three villages. Among the 39 WEPs used in more than one village, eight species were used with different local names. *Phyllanthus emblica* had different local names in all three villages.

### Use of WEPs

Although villagers collected WEPs for their own consumption, nine species, namely, *Acacia concinna*, *Acacia pennata* subsp. *kerrii*, *Archidendron jiringa*, *Cheilocostus speciosus*, *Lasia spinosa*, *Markhamia stipulata*, *Oroxylum indicum*, *Phyllanthus emblica* and *Telosma cordata*, were collected for selling at local markets.

A total of 47 species from 25 families were used as vegetables. All five species in Araceae were used as wild vegetables. Based on the interviews with villagers, the most preferred species for use as wild vegetables were *Phyllanthus emblica*, *Rotheca serrata* and *Docynia indica*. *P. emblica* and *R. serrata* were also used as medicinal plants in the study area. In addition to collecting it from the wild, the villagers planted *P. emblica* in their home gardens for their own consumption.

A total of 31 species from 23 families were used for their fruits and nuts. Species in Myrtaceae, Moraceae and Sapindaceae were frequently used as fruit plants. The most preferred fruit plants were *Docynia indica*, *Ficus semicordata*, *F. auriculata*, *Myrica esculenta* and *Archidendron jiringa*. In addition to collecting it from the wild, small-scale planting of *Docynia indica* was observed not only for fruits but also for fuel wood. The whole plants of *Oxalis latifolia* were eaten by children as a snack in Myin Ka village. This species is a perennial weed originating from Central and South America [35] and is growing in home compounds of the village. From the other two villages, no species were reported as a snack for children.

Four species from three families were used as spices and condiments in traditional curry. The dry powder of the rhizome from *Curcuma* cf. *amada* was used in various traditional dishes. Freshly pounded rhizome of *C.* cf. *amada* was also used as a spice. Leaves of *Cinnamomum tamala*, *Laurus* cf. *nobilis* and *Zanthoxylum armatum* were used in traditional curry for flavor. *Z. armatum* is also used as a spice in Yunnan, the province of China nearest to Myanmar [36].

The different methods of preparation of vegetables for traditional dishes are as follows: (i) *Hin*, the most common traditional food, is prepared by boiling the vegetable with edible oil, fermented fish and salt into a semiliquid dish; (ii) *Akyaw* is prepared by frying the vegetable with salt, spices, chili, and fermented fish; (iii) *Athote* is prepared by mixing the raw or boiled vegetable with pounded fried groundnut, salt and chili; (iv) *Atoe* is a raw salad eaten with fermented fish or with fermented bean; and (v) *Hincho* is a soup prepared by boiling the vegetable with fermented fish and salt.

Most of the WEPs in this study were used directly as vegetable dishes without any pretreatments, although it is known that some plant species, such as those in *Arisaema* and *Celastrus*, are toxic. Only the tubers of *Amorphophallus purpurascens* and *A.* cf. *muelleri* were boiled, peeled and pounded before preparing traditional dishes. Most of the fruits, which were consumed as a snack food, were eaten fresh without any preparation. The fresh fruits of *Ficus racemosa* were preserved by soaking them in salty water. The petiole of *Colocasia esculenta* and the fruits of *F. semicordata* were pickled and consumed as a fermented food similar to pickles.

### Use of WEPs as medicine

A total of 18 WEPs from 15 families were used as medicine (Table 3). Among these species, 11 were consumed as food for medicinal uses, and seven species were prepared as a main ingredient for traditional medicine. *Dichrocephala integrifolia*, *Plantago major* and *Laggera alata* were rarely used as food in the study area; however, these species were traditionally consumed for medicinal use. In the study area, the shoots and leaves of *D. integrifolia* were prepared as soup and consumed as a postpartum tonic for mothers. This species is also reported as a valuable medicinal plant for the treatment of Alzheimer’s disease [37]. The young leaves of *P. major* were traditionally prepared as salad and consumed for hypertension and food poisoning. It is reported that the extract from *P. major* has antifungal activity [38]. The shoots of *L. alata* were fried with eggs and consumed to treat swelling in the body. Isochlorogenic acid A from *L. alata* is reported as a potential candidate antihepatitis B drug [39].

**Table 3:** List of wild edible plants used as medicinal plants

Taxon	Use	Ailment
*Alternanthera philoxeroides*	Food as medicine	Inflamation
*Arisaema erubescens*	Food as medicine	Digestive system disorders
*Celastrus paniculatus*	Food as medicine	Nutritional Disorders
*Centella asiatica*	Ingredient for medicine	Respiratory system disorders; Sensory system disorders
*Cheilocostus speciosus*	Ingredient for medicine	Digestive system disorders
*Cinnamomum tamala*	Ingredient for medicine	Birth related disorders
*Colocasia esculenta*	Ingredient for medicine	Poison (Insect poison)
*Curcuma aromatica*	Ingredient for medicine	Injuries
*Curcuma* cf. *amada*	Ingredient for medicine	Digestive system disorders
*Dichrocephala integrifolia*	Food as medicine	Birth related disorders
*Laggera alata*	Ingredient for medicine	Inflamation; Pains; Injuries
*Macropanax dispermus*	Food as medicine	Digestive system disorders
*Momordica subangulata*	Food as medicine	Nutritional disorders
*Oroxylum indicum*	Food as medicine	Sensory system disorders
*Phyllanthus emblica*	Food as medicine	Circulatory system disorders; Respiratory system disorders
*Plantago major*	Food as medicine	Poison (Food poison); Skin/Subcutaneous/Cellular tissue disorders; Injuries; Circulatory system disorders
*Rotheca serrata*	Food as medicine	Digestive system disorders; Mental disorders
*Telosma cordata*	Food as medicine	Mental disorders

## Discussion

### Species identification

We applied the DNA barcoding technique as a guide for species identification. The chloroplast *rbcL* sequences of 71 species (85.5% of 83 identified species) were successfully sequenced. The sequences of 53 species (71.6% of sequenced species) yielded a single haplotype from a particular species from the BLAST analysis, whereas the remaining sequences yielded two or more haplotypes from closely related genera. As many of the collected specimens were sterile plants, the DNA barcoding technique facilitated the identification of the WEPs in this study.

### Notable uses of WEPs

**Fig. 3:**
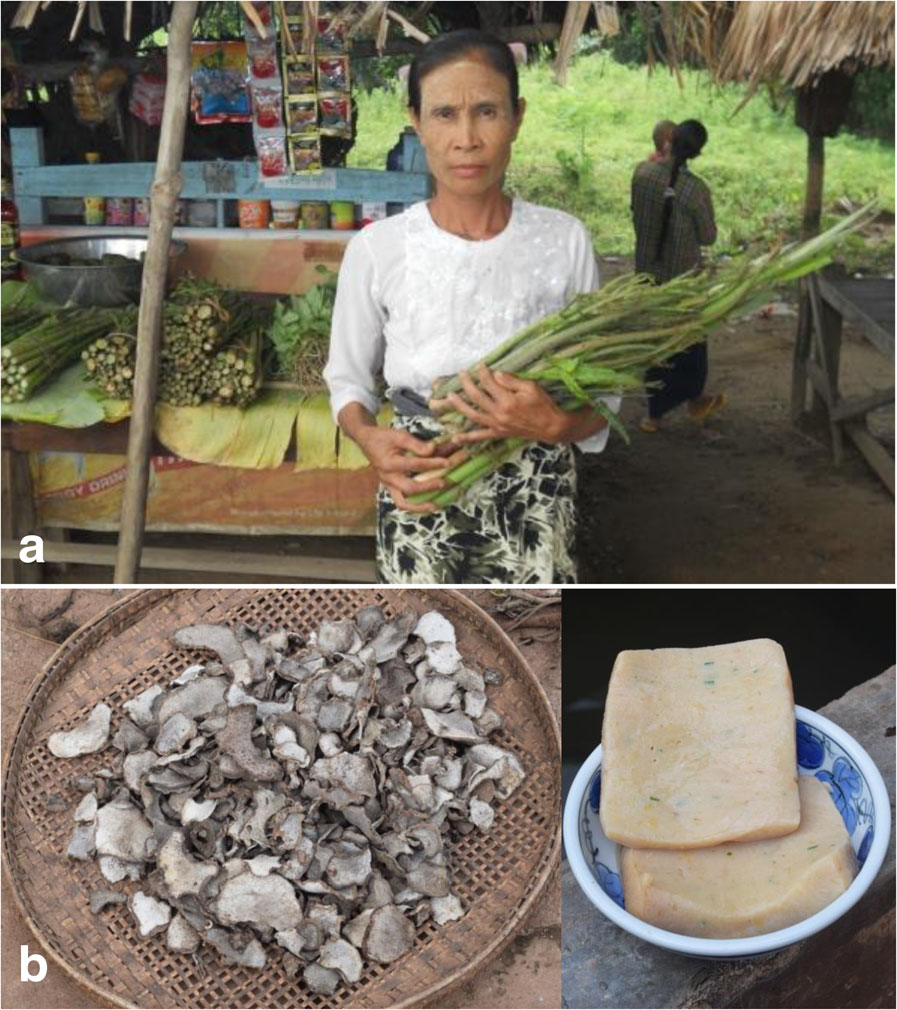
Use of WEPs in the study area. **a** wild *Lasia spinosa* and *Cheilocostus speciosus* at a local roadside market. Informed consent was obtained for the use of the photograph. **b** sun-dried slices of *Amorphophallus purpurascens* for preservation (left) and ‘Wa-u’ (right)

*Lasia spinosa* and *Cheilocostus speciosus* were important seasonal foods for the cash income of the local people. The villagers collected these two species from the wild seasonally and sold them in the local markets. Because customers prefer these two species as wild plants, the villagers do not cultivate them for markets (Fig. 3a). Tender leaves of *Acacia concinna* were used as vegetables, but the decoction of fruits of *Acacia concinna* is traditionally and popularly used as a shampoo in Myanmar. The stems and tubers of *Amorphophallus purpurascens* and *Amorphophallus* cf. *muelleri* were used as vegetables, and the sliced tubers were preserved after being sun dried. The tubers of these species were boiled, peeled and pounded to make konjac, referred to as ‘Wa-u’ in Myanmar (Fig. 3b). Boiled seeds of *Archidendron jiringa* are a traditional food in the area, and the seeds are also a source of income for the villagers. Although the boiled seeds are consumed widely in the tropics, they have also been found to have some toxicological effects on the heart, kidney, liver and pancreas [40].

The seeds of *Elaeocarpus floribundus* were used to extract vegetable oil in Myin Ka village. The vegetable oil was used locally, but it was not produced at a commercial scale. To the best of our knowledge, there are no reports on the use of seed oil of *E. floribundus*, although its fruits are eaten raw as a wild edible fruit in South Asia [41]. It is interesting that the extracts of leaves of *E. floribundus* showed significant activities against CEM-SS cancer cells [42].

It is reported that *Rotheca serrata* is mainly used as a medicinal plant in other areas [43]. However, *R. serrata* was mainly consumed as a wild vegetable in the study area and was used for digestive system disorders. The young leaves of *Celastrus paniculatus* were prepared in a traditional soup and consumed as a dietetic food. The food is not used for a particular ailment. However, the villagers believe that the soup is good for health. The use of this species was recorded in a traditional song expressing that the plant is highly recommended for traditional soup. The fruits of *C. paniculatus* are consumed orally as a vermifuge by the locals living around Popa Mountain Park, Myanmar [44]. It was reported that the extract from *C. paniculatus* can inhibit the growth of breast cancer cells [45].

### WEPs used in each village

The differences in the use of WEPs between the communities of three villages have been observed. The differences among ethnic groups could be the main reason for using different local names for the same species. The three villages are situated in different townships, and there are no social relationships among the villages. This is also another reason for using different local names.

The village of Myin Ka used a larger number of WEPs than the other two villages. Myin Ka village has a longer history than the other two villages and could have inherited much more traditional knowledge of WEPs. In spite of the same major ethnic group (*Danu* people) and similar vegetation being found in the villages of Myin Ka and Pin-sein-pin, 33 out of 71 WEP species were used in both of the villages, but the remaining 38 species were not common between the two villages. These two communities have different historical backgrounds, and there were no social relationships between the two villages. These differences may be a cause of different uses of the plant resources.

The village of Eden has fewer shared species with the other two villages. Most of the species documented in this study from the families Ebenaceae, Elaeagnaceae, Elaeocarpaceae, Fagaceae, Lamiaceae, Lauraceae, Myricaceae, Rosaceae, Rubiaceae and Rutaceae were used in the villages of Myin Ka and Pin-sein-pin, but not in the village of Eden. However, most species from the families Burseraceae, Lythraceae and Sapindaceae were used only in Eden village. The elevation of Eden is approximately 350 m above sea level, while that of Myin Ka and Pin-sein-pin is more than 1000 m. The forests around the village of Eden are mostly deciduous forests, while those around the villages of Myin Ka and Pin-sein-pin are evergreen forests. These differences could be the causes of variation in plants used by the local communities. Moreover, the village of Eden is newly established by *Kayan* people who migrated from Kayah State, which is different from the villages of Myin Ka and Pin-sin-pin, which were established by *Danu* people in Shan State. The migrant community may be less familiar with the plant resources in the new environment. In addition, the villagers of Eden practice shifting cultivation for banana plantations. Shifting cultivation could have detrimental effects on the environment, and the availability of WEPs could be diminished.

The average number of species recognized by a single key informant was different among the three villages (Table 4). The key informants from Myin Ka village reported a greater number of species (an average of 18.0 species per key informant) than did those from the villages of Eden and Pin-sein-pin (an average of 10.2 and 12.1 species, respectively). The amount of knowledge on WEPs was also different between the age classes of key informants in the study area. Usually, the older generations transfer the farming activities and knowledge of WEPs to the younger generations in the study area. However, the age class of 30–39 years reported more WEPs than did the older groups. The interviews revealed that the age class of 30–39 years was much more familiar with the environments where they could collect WEPs. The sharing of work between family members was revealed, and the age class of 30–39 years took more responsibilities in the collection of WEPs. The older generations might face the inability to recall the knowledge of gathering WEPs. The abundance of knowledge of WEPs in younger age classes and a decrease in the knowledge exhibited by the older groups have been reported previously [46, 47].

**Table 4:** The numbers of wild edible plants recognized by each key informant

Age	Eden		Myin Ka		Pin-sein-pin	
	Informant	No. of WEPs recognized	Informant	No. of WEPs recognized	Informant	No. of WEPs recognized
30–39	A	12	F^a^	21	M	22
			G	28	N	7
			H	30		
40–49	B	11	I	12	O	8
	C	17	J	13	P	9
	D	5			Q	10
50–59	E	6	K	9	R	18
			L	13	S	11
Mean ± SD		10.2 ± 4.9		18.0 ± 8.4		12.1 ± 5.6

^a^: female

The number of species reported as WEPs by more than two key informants in the villages of Eden, Myin Ka and Pin-sein-pin was 8 (33.3% of all species reported by all key informants in Eden), 29 (56.9%) and 15 (40.5%) species, respectively. This result indicates more share knowledge of WEPs within the community of Myin Ka. Myin Ka village has 120 ha of common land for watershed conservation, and all villagers of Myin Ka village have access to the common land for collection of WEPs. In contrast, most of the land area around Pin-sein-pin village is privately owned. In the case of Eden village, shifting cultivation is practiced in the reserve forests, where the villagers do not have the legal right of land ownership. Thus, land use and the land ownership system could have effects on the sharing of knowledge of WEPs among the villagers.

## Conclusion

The rich diversity and traditional knowledge of WEPs have been documented in this study. This study indicates that WEPs play an important role in the livelihood of local communities. Historical background, land use system and surrounding vegetation could have effects on the variation in the traditional uses of WEPs. The indigenous societies, especially that in the village of Myin Ka, maintained a considerable amount of traditional knowledge of WEPs. In addition to their food value, other uses of WEPs, such as their medicinal uses, make them more important in the livelihood of local people. WEPs are important not only for increasing the diversity of local food consumption but also for generating income in the local communities. Increasing awareness of the importance of WEPs will encourage the conservation of traditional knowledge of indigenous populations. Further investigations on the nutritional value and pharmacological activities of WEPs will add more value to the traditional knowledge.

## Abbreviations

**MBK**: Makino botanical garden
**WEP**: Wild edible plant
